# Assessment of Breast Cancer Mortality Trends Associated With Mammographic Screening and Adjuvant Therapy From 1986 to 2013 in the State of Victoria, Australia

**DOI:** 10.1001/jamanetworkopen.2020.8249

**Published:** 2020-06-23

**Authors:** Robert Burton, Christopher Stevenson

**Affiliations:** 1 School of Public Health and Preventive Medicine, Monash University, Melbourne, Australia; 2 School of Health and Social Development, Deakin University, Geelong, Australia

## Abstract

**Importance:**

Diagnosis of early breast cancer (EBC) in women by mammographic screening and postsurgical adjuvant endocrine therapy and chemotherapy (termed adjuvant therapy) began simultaneously in many countries in the 1990s. Subsequent breast cancer mortality declines were variously attributed to mammographic screening and/or adjuvant therapy.

**Objective:**

To determine the relative mortality reductions associated with these 2 interventions in women with EBC who had been exposed to both.

**Design, Setting, and Participants:**

This secondary analysis of cross-sectional studies assessed groups of women with invasive breast cancer in the State of Victoria, Australia, from January 1, 1982, to December 31, 2013, who were included in the Victorian Cancer Registry (VCR). The population consisted of participants in population-based studies of female breast cancer from 1986 to 2013 using data from 4 VCR population-based surveys of breast cancer treatment from 1986 to 1999; VCR data on breast cancer incidence, mortality, and TNM stage at diagnosis from 1986 to 2013; and Victorian mammographic screening program (BreastScreen Victoria) data from 1992 to 2007. Breast cancer incidence and mortality data were analyzed for all 76 630 women registered with invasive breast cancer with the VCR from January 1, 1982, to December 31, 2013, and breast cancer treatment and screening data were analyzed additionally for the groups of surveyed women as described above.

**Exposures:**

Participation in BreastScreen Victoria and receipt of adjuvant therapy after surgery for EBC.

**Main Outcomes and Measures:**

Data were analyzed for associations between crude breast cancer mortality trends and uptake of adjuvant therapy and downstaging by mammographic screening.

**Results:**

Of all 76 630 women registered with breast cancer with the VCR from January 1, 1982, to December 31, 2013. Joinpoint analyses of the time trend in crude mortality showed an increase from 31.6 per 100 000 women in 1982 to 34.3 per 100 000 women in 1994, with a single joinpoint at 1994, followed by a significant declining trend to 23.9 per 100 000 women in 2013 (annual percentage change, −1.3%; 95% CI, −1.6% to −0.9%). By 1999, 74% of all Victorian women with EBC (737 of 1001) had commenced adjuvant endocrine therapy, and 72% (187 of 260) of premenopausal and 29% (215 of 741) of postmenopausal women with EBC had commenced adjuvant chemotherapy. Crude incidence of advanced-stage breast cancer almost doubled from 12.2 per 100 000 women in 1986 to 23.9 per 100 000 women in 2013.

**Conclusions and Relevance:**

This study found that mammographic screening did not downstage breast cancer in Victoria from advanced to early, so population mortality benefit is lacking. Adjuvant therapy uptake was associated with all of the decline in Victorian breast cancer mortality since 1994. Given these findings, monitoring the relative contributions of mammographic screening and adjuvant therapy for EBC to breast cancer mortality reductions in populations of women exposed to both should be mandatory.

## Key Points

**Question:**

Is population-based mammographic screening or endocrine therapy and chemotherapy (adjuvant therapy) after curative surgery for early or operable breast cancer associated with the decline in breast cancer mortality in Victoria, Australia?

**Findings:**

In this secondary analysis of cross-sectional studies of breast cancer mortality, stages at diagnosis and adjuvant therapy uptake were compared in Victoria. Advanced breast cancer incidence doubled from 1986 to 2013, and crude breast cancer mortality declined by 30% after 1994; by 1999, most women were receiving adjuvant therapy, which may be associated with this decline.

**Meaning:**

These findings suggest that adjuvant therapy, rather than mammographic screening, is associated with the decline in breast cancer mortality in Victoria, Australia.

## Teaser

This secondary analysis of cross-sectional studies assesses the relative mortality trends associated with mammographic screening and postsurgical adjuvant endocrine therapy and chemotherapy among women with early breast cancer in Victoria, Australia.

## Introduction

Since 1988, the Early Breast Cancer Trialist Collaborative Group (EBCTCG) has been conducting systematic reviews of the effects of endocrine therapy and cytotoxic chemotherapy (adjuvant therapy), administered after surgical removal, on mortality due to breast cancer confined to the breast and adjacent axilla (early breast cancer [EBC]).^1,2^ The fifth EBCTCG review in 2005^2^ reported that, throughout the next 15 years, breast cancer–specific mortality in women with EBC would be approximately halved by 6 months of anthracycline-based chemotherapy followed by 5 years of adjuvant tamoxifen therapy and, for middle-aged women with estrogen (ER)-positive cancer, administering this adjuvant chemotherapy to premenopausal women for more than 1 year and adjuvant tamoxifen to all women for more than 2 years would significantly reduce their cumulative mortality due to breast cancer.^2^

To reduce breast cancer mortality by screening, cancer must be detected at an early stage when cure by treatment is possible.^3^ Therefore, monitoring mammographic screening in populations should include measurement of the stages at diagnosis to determine whether screening is reducing the incidence of advanced breast cancer, defined by the American Joint Cancer Commission (AJCC) as stage III breast cancer locally invasive beyond the breast and/or with metastatic breast cancer in fixed axillary lymph nodes and any lymph node metastases in regional nonaxillary lymph nodes or as stage IV breast cancer with hematogenous metastases to organs and tissues distant to the breast,^4^ compared with EBCTCG EBC,^2^ which is AJCC stages I and II (downstaging).^3^ Unfortunately, analyses of long-term trends of advanced breast cancer incidence for decades are only available today for populations screened by mammography in New South Wales (NSW), Australia,^5^ the United States,^3^ Norway,^6^ and the Netherlands.^7^ All 4 countries reported that advanced breast cancer incidence either remained stable^3,6,7^ or increased^5^ after mammographic screening began, so no downstaging to EBC was detected.

A 2012 analysis of age-specific breast cancer incidence and mortality trends from 1986 to 2007 in Australia concluded that most of the 28% decline in age-specific breast cancer mortality since 1994 could be attributed to adjuvant therapy uptake by women with EBC, not to the Australian mammographic screening program (BreastScreen).^8^ Stage-specific crude incidences of female breast cancer in the State of Victoria are now available from 1986 to 2013, so the objective of this study was to compare their trends with trends in adjuvant therapy uptake in the female population from 1986 to 1999 and trends in breast cancer mortality from January 1, 1982, to December 31, 2013.

## Methods

This secondary analysis of cross-sectional studies compares trends in breast cancer mortality among Victorian women, stage at diagnosis, and uptake of adjuvant therapy by women with EBC. Each individual study met the Strengthening the Reporting of Observational Studies in Epidemiology (STROBE) reporting guideline when published.^9^ This report follows the STROBE guidelines for discussion, including presentation of key results, study limitations, interpretation, and generalizability. All of the epidemiological data in this report are in the public domain and have been published and/or can be obtained by request to cancer registries or calculated from publications^10,11,12,13,14,15^ (eMethods 1 in the Supplement) using Australian Bureau of Statistics data.^16^ Ethics permission was sought and obtained as necessary by the original studies.^4,10,11,12,13,14,15^ The present study was exempted from review post hoc by the Deakin University Human Research Ethics Committee for the use of deidentified data and meeting the definition of negligible-risk research. This study followed the ethical principles of the Declaration of Helsinki.^17^

In Australia since 1982, all new cases of cancer (invasive malignant neoplasia), except basal and squamous cell skin cancers, have been registered by law with the 8 state and territory cancer registries.^8^ By 2000, 10 population-based management studies (treatment surveys) of female breast cancer were undertaken in Australia, including 9 state-based and 1 national survey in 1995,^4,10,11,12^ most of which focused on operable breast cancer, which is AJCC stages I and II EBC.^4^ The national survey also reported the survey methods in detail and a summary of 7 of the state surveys.^4^

**Figure 1.**
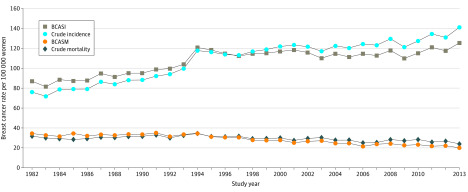
Incidence of Breast Cancer and Mortality Trends in Victoria, Australia: 1982-2013 BCASI indicates breast cancer age-standardized incidence; BCASM, breast cancer age-standardized mortality.

For this study based in Victoria, relevant data were obtained directly and/or by calculation from all 3 state surveys and the Victorian component of the national surveys on adjuvant therapy commencement (uptake) for all premenopausal (or younger than 50 years) and all postmenopausal (or 50 years or older) women with EBC and the TNM stage at diagnosis for all women diagnosed with breast cancer in 1986, 1990, 1995, and 1999.^10,11,12^ Crude Victorian data on breast cancer stages at diagnosis were published for 2006 and 2007^12^ and subsequently for 2006 to 2013 in the Victorian Cancer Registry (VCR) 2014 annual Cancer in Victoria report.^13^ This 2014 report stated that the proportions of breast cancer stages were stable from 2006 to 2013. However, crude breast cancer incidence rose progressively during that period^13^ (Figure 1), so the years 2006, 2007, 2010, and 2013 within that period were also used for staging. Of note, Victorian age-standardized staging data have never been published.^13^

We used the above staging data to calculate trends of crude incidence of Victorian stages I and II EBC and advanced stages III and IV (III/IV) breast cancer from 1986 to 2013 (Table 1).^16^ These crude incidences were all corrected for missing data to 16% of women in 1986 (eMethods 1 in the Supplement), 11% in 1990, 4% in 1995, 9% in 1999, 4% in 2006 to 2007, and 6% in 2006 to 2013^10,11,12,13,14,15^ by assuming that the proportions of Victorian women with various stages of breast cancer in the missing data were the same as those for whom data were available. Of note, missing data were only 4% in 1995 and 6% in 2006 to 2013, respectively,^12,14^ so sensitivity analyses to test missing data assumptions were not indicated.

**Table 1.** Crude Incidence Rates for Breast Cancer Stratified by Early Cancer and Stage

Survey	Total No. of cases	Early breast cancer, No. (%)	Stage I		Stage II		Stages III/IV	
			No. (%)	Crude incidence rate	No. (%)	Crude incidence rate	No. (%)	Crude incidence rate
Victoria 1986^a^	799	671 (84)	436 (55)	41.5	235 (29)	22.4	128 (16)	12.2
Victoria 1990^a^	NA	856 (NA)	471 (55)^b^	42.8	265 (31)^b^	24.1	NA	NA
Victoria 1995^a^	1316	1158 (88)	776 (59)	67.5	382 (29)	33.2	158 (12)	13.7
Victoria 1999^a^	NA	1181 (100)	744 (63)	62.0	437 (37)	36.4	NA	NA
Staging project 2006^c^	2974	2500 (84)	1407 (47)	54.2	1093 (37)	42.2	474 (16)	18.3
Staging project 2007^c^	2990	2466 (82)	1343 (45)	50.7	1123 (38)	42.8	524 (18)	19.2
Victoria 2010^c^	3499	2904 (83)	1645 (47)	58.7	1260 (36)	45.0	595 (17)	21.3
Victoria 2013^c^	4075	3382 (83)	1915 (47)	66.0	1467 (36)	50.6	693 (17)	23.9

Abbreviation: NA, not available.

^a^ Annual crude incidences are calculated from 6-month data.^10,11,12^

^b^ The 1990 proportions are those available for early breast cancer and are not corrected for missing data because the total registered was not reported.

^c^ Annual crude incidences are calculated from annual data.^13,14,15^

Breast cancer mortality rates are derived from data collected from death certificates and validated by VCR staff.^10,14^ The crude incidence and mortality rates of Victorian female breast cancer from 1982 to 2013 were provided by the VCR (eTable in the Supplement), and published age standardized to year 2001 breast cancer incidence (BCASI) and mortality (BCASM) rates from the VCR were also used^14^ (eTable in the Supplement). The relative reduction in breast cancer mortality between any 2 periods was calculated as the difference in the mortality rate between the 2 periods divided by the mortality rate for the first period.^8^

The 1995 national survey reported that 25% of Australian women 20 years or older resided in Victoria in 1995 and that more than 90% of breast cancer in 1995 was diagnosed in women 40 years or older.^4^ In 2011, 25% of Australia’s 11.2 million women (2.8 million) resided in Victoria.^16^

The Australian government offers 2 yearly free population-based mammographic screenings for women 40 years or older, with women aged 50 to 69 years invited biennially (BreastScreen).^18^ The BreastScreen program, funded in 1991, is implemented by the 6 state and 2 territory governments. The Victorian component, BreastScreen Victoria, was developed from 1 regional service with 14 screening sites in 1991 to 8 regional services with 59 screening sites in 1995 and reported on its first decade of screening (1992-2002) in 2003.^18^ Participation in BreastScreen Victoria by women aged 40 to 79 years increased from 4.8% in 1992 to 1993 to a plateau of approximately 37% from 1998, which was also the national participation rate; that rate continued to 2010.^18,19^ For the women aged 50 to 69 years who were invited, participation increased from 48.3% in 1994 to 1995 to 57.5% in 1996 to 1997^18^ and then plateaued at the national level of 53% to 57%.^18,19^ From 5% to 10% of Victorian women were undergoing mammographic screening outside BreastScreen before 1992 and after.^8,18^ Rescreening responses to the biennial invitation are a BreastScreen quality indicator; the 2005-2006 BreastScreen Australia report shows that approximately 60% of Victorian women who attend a first screening attend a second screening, and 80% of those women attend third and subsequent mammographic screenings.^20^

### Statistical Analysis

Data were analyzed from January 1, 1982, to December 31, 2013. Joinpoint analysis of the crude breast cancer mortality trend was undertaken using version 4.8.0.1 of the software developed by the US National Cancer Institute.^21^ This analysis objectively identifies (by permutation analysis) joinpoints as those points where a significant change in the linear slope of the mortality trend (log scale) was detected during the study period^22^ and presents the segments of the mortality trend on each side of a joinpoint as annual percentage changes (APCs) with 95% CI.^21^ This presentation allows identification of statistically significant changes in the mortality trend and, by identifying the year of the change, associates these changes with interventions, such as the introduction of screening or adjuvant therapy. The joinpoint analyses used 2-sided hypothesis tests with *P* = .05 as identifying statistical significance.^21,22^

## Results

These analyses include breast cancer data collected by law in the VCR for all 76 630 women registered with invasive breast cancer in Victoria from 1982 to 2013; treatment and stage at diagnosis from 4 published VCR-based surveys of 6 months each in 1986, 1990, 1995, and 1999; and stage at diagnosis from all women registered with the VCR from 2006 to 2013. Breast cancer crude incidence and BCASI rates increased from 1982 to 2013, and the corresponding crude mortality and BCASM rates decreased (Figure 1). The crude incidence rate increased by 100 per 100 000 population from 1986 and 1992 and then in 1994 peaked at 120 per 100 000 above the 1982-1992 trend, declining to a fluctuating increasing trend without a subsequent compensatory drop below the 1986-1992 trend (Figure 1), as shown by fitting a linear trend to the data for 1982 to 1991 by joinpoint and using it to extrapolate to 2013, giving predicted values of 73.85 in 1982 and 127.97 in 2013,^21^ evidence of overdiagnosis of EBC based on mammographic screening.^3^

**Figure 2.**
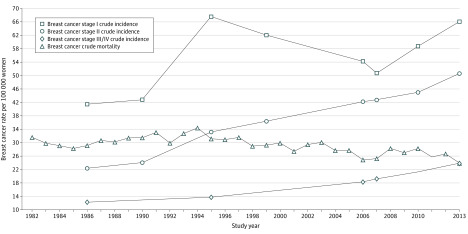
Crude Mortality Due to Breast Cancer and Stage Incidence Trends in Victoria, Australia: 1982-2013

Joinpoint analyses of the time trend in crude mortality showed that annual crude mortality increased from 1982 to 1994, but this increase was not statistically significant (APC, 0.7%; 95% CI, −0.1% to 1.5%). A single joinpoint occurred at 1994 (best-estimate 95% CI, 1990-1997), followed by a significant declining trend to 2013 (Figure 2) (APC, −1.3%; 95% CI, −1.6% to −0.9%), a relative reduction in breast cancer mortality of 30% since 1994 (Figure 1). Importantly, there is no second joinpoint in this crude mortality trend, so no evidence of a statistically significant association of mammographic screening with the Victorian breast cancer mortality decline after 1994 was found.

The crude incidence of stage I breast cancer in Victoria increased by 3% from 1986 to 1990 (Table 1 and Figure 2). This initial increase was followed by a large increase of 62% from 1990 to 1995, when 30% or more of Victorian women 40 years or older were screened.^18^ Victorian stage II crude incidence more than doubled after rising above the 1986-1990 trend. Crude incidence of advanced stages III/IV breast cancer increased by 96% from 12.2 to 23.9 per 100 000 women from 1986 to 2013, ruling out a direct association of mammographic screening with breast cancer mortality (Table 1 and Figure 2).

In 1986 in Victoria, only 127 of 635 women (20%) with EBC began endocrine therapy, which consisted of tamoxifen in 94% to 100% of women. However, this proportion had almost doubled by 1990 (298 of 764 [39%]) and more than tripled by 1999 (737 of 1001 [74%]) (Table 2). Adjuvant chemotherapy uptake for premenopausal women increased from 40 of 190 (21%) in 1986 to 187 of 260 (72%) in 1999 and for postmenopausal women, from 20 of 445 (4%) in 1986 to 215 of 741 (29%) in 1999 (Table 2). By 1995, the standard combination adjuvant therapy of combined cyclophosphamide, methotrexate sodium, and fluorouracil had changed to a combination of cyclophosphamide and the anthracycline adriamycin.^12^ The crude breast cancer mortality peaked from 31.6 per 100 000 women in 1982 to 34.3 per 100 000 women in 1994 (Figure 2) and then fell continuously to 23.9 per 100 000 women in 2013. In 1995, 57% of premenopausal women began adjuvant chemotherapy and 62% of all women with EBC began tamoxifen, and in 1999, adjuvant premenopausal chemotherapy uptake had increased to 72% and adjuvant tamoxifen uptake had increased to 74% (Table 2), consistent with the EBCTCG findings that adjuvant therapy uptake could decrease breast cancer mortality after 1 year.^2^

**Table 2.** Victorian Population Surveys of Adjuvant Therapy Uptake by Women With Early Breast Cancer^10,11,12^

Study year	Adjuvant therapy uptake, No./total No. (%)				All women, tamoxifen uptake, No./total No. (%)
	Premenopausal women or younger than 50 y		Postmenopausal women or 50 y or older		
	Tamoxifen	Chemotherapy	Tamoxifen	Chemotherapy	
1986	11/190 (6)	40/190 (21)	116/445 (26)	20/445 (4)	127/635 (20)
1990	NA	NA	NA	NA	298/764 (39)
1995	58/235 (25)	134/235 (57)	599/832 (72)	141/832 (17)	657/1067 (62)
1999	159/260 (61)	187/260 (72)	578/741 (78)	215/741 (29)	737/1001 (74)

Abbreviation: NA, not available.

Adjuvant therapy uptake for EBC in Victoria increased more than 3-fold from 1986 to 1999 (Table 2). When analyzed using EBCTG 2005 data for adjuvant tamoxifen therapy^2^ and EBCTCG 2012 data for adjuvant anthracycline and cyclophosphamide therapy^23^ as described previously^7^ (eMethods 2 in the Supplement), the 1999 adjuvant therapy uptake (Table 2) can account for the entire 30% decline in Victorian crude breast cancer mortality after 1994. By comparing the crude mortality trend from 1984 to 1994 extrapolated by the 95% CI bound of the APC of 1.5% (above) with the observed trend, we calculated that as many as 3688 Victorian women could have had their deaths due to breast cancer prevented by this adjuvant therapy from 1994 to 2013 (eTable in the Supplement).

## Discussion

A 2013 Victorian VCR–based cohort study indicated that most women who begin oral adjuvant endocrine therapy persist with it.^24^ Using the same calculation strategy (eMethods 2 in the Supplement) and the national survey data,^4^ the adjuvant therapy that Australian women with EBC commenced in 1995 may account for 23% of the observed 28% mortality decline in breast cancer nationally and for all of the decline if the 1999 Victorian adjuvant therapy data are used (Table 2).^12^ In Western Australia, crude breast cancer mortality fell by 24% from 1999 to 2014.^25^ Using this same calculation strategy (eMethods 2 in the Supplement), the adjuvant therapy that Western Australian women with EBC commenced in 1999^26^ could account for all of that mortality decline. Furthermore, axillary lymph node metastases in women with EBC in Western Australia increased by more than 70% in postmenopausal women with ER-positive EBC from 1990 to 1999,^26^ indicating that mammographic screening was not associated with reduced stages II and III breast cancer incidence in that decade.

In the 1999 Victorian management survey, with approximately one-third of women aged 50 to 69 years diagnosed via BreastScreen Victoria,^12^ the proportions of women with ER-positive breast cancer commencing adjuvant tamoxifen therapy were almost the same for women diagnosed clinically as via BreastScreen (95% vs 92%). For adjuvant chemotherapy uptake, the proportions were similar for women with ER-negative and ER-positive cancer (71% vs 64%) (Vicki White, PhD; unpublished data from the 1999 survey; received by email November 1, 2019). However, more women with ER-positive cancer diagnosed clinically commenced adjuvant chemotherapy than women diagnosed via BreastScreen (39% vs 22%). Therefore, no evidence suggests that diagnosis via BreastScreen was associated with greater access to adjuvant therapy and subsequently reduced breast cancer mortality. Unfortunately, often overlooked in evaluating mammographic screening is the extent to which improvements in adjuvant therapy given after surgery for EBC have reduced the potential of screening mammography to have a direct effect on breast cancer mortality.^27,28^

A 2016 International Agency for Research on Cancer systematic review of breast cancer screening for 72 countries,^29^ where more than half of all of the world’s women live, reported that screening mammography was available to some or all populations of women. However, no trend data for advanced-stage breast cancer were reported for any country,^29^ so the effectiveness of that screening cannot be evaluated from that report. A 2018 systematic review of the effect of mammographic screening on advanced breast cancer incidence in Europe^30^ could not reach a conclusion because, together with other problems, staging did not have a consistent definition and was not ascertained consistently. A systematic review of 8 of the original randomized clinical trials of population mammographic screening vs controls for whom staging data were available^31^ found that the effect of early detection on reducing the incidence of advanced-stage breast cancer accounted for most of its effect on breast cancer mortality and that monitoring the incidence of advanced-stage breast cancer as an early indicator of the effect of service screening was indicated.

The age-standardized incidence of advanced metastatic breast cancer (Surveillance, Epidemiology, and End Results distant disease^3^) for women in NSW, Australia’s most populous state (3.6 million women in 2011), increased by 67% from 4.3 to 7.2 per 100 000 population from the 1988-1995 to the 2006-2012 periods,^5^ suggesting that mammographic screening was not downstaging breast cancer in that state. Crude Australian data for advanced breast cancer (AJCC stages III/IV) are available for 1995 (1210 of 10 081 women [12%])^4^ and 2011 (2403 of 14 569 women [17%]),^32^ resulting in calculated annual crude national incidence rates of stages III/IV breast cancer of 13.3 per 100 000 women in 1995 and 21.7 per 100 000 women in 2011, a 63% increase. These NSW and Australian increases in advanced-stage cancer are consistent with the 74% Victorian increase in incidence of advanced stages III/IV crude breast cancer from 1995 to 2013 (Table 1 and Figure 2). For metastatic stage IV breast cancer in Australia (1995: 403 of 10 081 women [4%]^4^; 2011: 583 of 14 591 women [4%]^32^), there was a 45% increase per 100 000 population. Therefore, mammographic screening also appears to have failed to downstage breast cancer nationally. The reasons for these increases in advanced-stage breast cancer incidence in NSW, Victoria, and Australia are not known; however, Australian state and national all-stage incidences are increasing, and Australia now has the third highest age-standardized incidence rate in the world after Europe and North America (Figure 1).^29^ These increases are cause for global research efforts.^29^

In 1999, 69% of all Victorian women with EBC, 64% of women with EBC less than 1 cm in diameter, and more than 80% of women with EBC of 1 cm or more in diameter received postoperative external beam radiotherapy (EBRT).^12^ The potentially fatal cardiac adverse effects of EBRT to the breast have been recognized for decades,^27,33^ so these women were at risk of premature death without any possible benefit of reduced mortality.

The international, multicenter EBC TARGIT-A randomized clinical trial,^33^ in which the Western Australian female population participated, compared single-dose targeted intraoperative radiotherapy with fractionated daily EBRT after curative surgery. Overall breast cancer mortality was similar between the 2 groups after 5 years of follow-up, but there were significantly fewer deaths related to non–breast cancer causes with intraoperative radiotherapy (1.4% [95% CI, 0.8%-2.5%] vs 3.5% [95% CI, 2.3%-5.2%]; *P* = .009), attributable to fewer deaths due to cardiovascular causes and other cancers.^33^ When intraoperative radiotherapy was given concurrently with lumpectomy, compared with women receiving EBRT, there was a potential 2.3% decrease in overall mortality at 5 years.^33^ In 1999 in Victoria, 69% of the 1342 women with EBC received postoperative EBRT,^12^ so there could have been an extra 54 deaths in 2004, increasing to 78 deaths 5 years after 2013 if the proportions of women receiving EBRT had remained the same after 1999. Using data of 30% overdiagnosis of women aged 50 to 69 years in the NSW BreastScreen program,^35^ in 2012, we calculated an Australian ratio of harm of overdiagnosis to benefit (breast cancer deaths avoided) of 15:1 and recommended stopping the invitation to screening.^34^

### Limitations

This secondary analysis of cross-sectional observational data examining time trends across the study period can only show associations among Victorian breast cancer mortality, mammographic screening participation, and adjuvant therapy uptake. We can only infer causality by showing that these associations are unlikely to be compatible with a causal relationship between screening and a fall in mortality and are likely to be compatible with the uptake of adjuvant therapy after curative surgery for EBC.

## Conclusions

This analysis of cross-sectional studies showed no downstaging of breast cancer by mammographic screening, which suggests that persistence with BreastScreen Victoria may continue to expose Victorian women to unnecessary morbidity and mortality. We found that adjuvant therapy accounted for the observed 30% mortality decline; given this finding, we propose that BreastScreen should be terminated. Continuous measurement of breast cancer stages at diagnosis, all-cause and breast cancer–specific mortality, and adjuvant therapy uptake should be mandatory in monitoring and evaluating mammographic screening programs.

## Notes

Supplement. **eMethods 1.** Data Collection **eMethods 2.** Calculations **eTable.** Data Provided by Vicky Thursfield From VCR **eReferences.**
