# Errors in Results Section, References, and Figure Citation

**DOI:** 10.1001/jamanetworkopen.2020.14787

**Published:** 2020-07-09

In the Original Investigation titled “Assessment of Breast Cancer Mortality Trends Associated With Mammographic Screening and Adjuvant Therapy From 1986 to 2013 in the State of Victoria, Australia,”^1^ published online June 23, 2020, there was an error in the data presented in the first paragraph of the Results section; some references and a figure citation in the Results were also misnumbered. This article has been corrected.^1^
