# Supplementary material for: Assessment of Breast Cancer Mortality Trends Associated With Mammographic Screening and Adjuvant Therapy From 1986 to 2013 in the State of Victoria, Australia
Source: JAMA Netw Open. 2020 Jun 23;3(6):e208249. doi: 10.1001/jamanetworkopen.2020.8249 (PMC7312387; doi:10.1001/jamanetworkopen.2020.8249)
Supplement: Supplement. — eMethods 1. Data Collection eMethods 2. Calculations eTable. Data Provided by Vicky Thursfield From VCR eReferences. [file jamanetwopen-3-e208249-s001.pdf]

Supplementary Online Content

Burton R, Stevenson C. Assessment of breast cancer mortality trends associated with mammographic screening and adjuvant therapy from 1986 to 2013 in the state of Victoria, Australia. *JAMA Netw Open*. 2020;3(6):e208249. doi:10.1001/jamanetworkopen.2020.8249

eMethods 1. Data Collection

eMethods 2. Calculations

eTable. Data Provided by Vicky Thursfield From VCR

eReferences.

This supplementary material has been provided by the authors to give readers additional information about their work.

©2020 Burton R and Stevenson C. *JAMA Network Open*.

eMethods 1. Data Collection

Calculation of breast cancer stages from the Victorian 1986 survey[1]

**Results**		**799 total cases**	
Minus 22 registered too late	777		
Minus 20 no Vic surgeon	757		
So 42 of 799 have missing data			
		Advanced/Late Cancers	
Minus 33 advanced inoperable	724		33
Minus 8 operable/no surgery	716		

So 716 forms sent to surgeons for surgical data

Minus 81 forms not returned forms (11%)	635 data reported		
Total missing data all stages: 42 + 8+ 81 = 131 (16%)*			
Pathological stage 3 for 593 of 635: Table 2			9%
Corrected for 635 × 9/100			57
Plus surgeon preoperative metastatic: 3% of 635		19	
Subtotal inoperable on Survey forms returned=		76	

Correct inoperable for non-returns:

76 × 716/635 = 86

Plus 33* inoperable = 119 advanced/late for 757 cases=15·7%

Assume same proportions for missing data

So late stages 3/4 rounded = 799 × 15·7/100 = 125 (16%)

So EBC stages 1/2 rounded = 799-125/100 = 674 (84%)

©2020 Burton R and Stevenson C. *JAMA Network Open*.

eMethods 2. Calculations

The female mortality reductions between 1994 and 2013 in Australia in 1995 and Victoria and WA in 1999, which adjuvant therapy commenced by women diagnosed with EBC in 1995 and 1999 could have produced. ER+ Proportions for Victoria and WA from the 1995 National survey[2], since 1999 was EBC only. EBCTCG adjuvant Tamoxifen and chemotherapy references[3,4] used for calculations

AUSTRALIA 1995: all data from the NBCC survey[2]

All women Tamoxifen Therapy

88%* (proportion of all breast cancer that was EBC) × 59% (proportion of women with EBC who were ER+ and received tamoxifen[2]) × 31% (EBCTCG: adjuvant tamoxifen reduction in mortality for ER+ women aged 40-70 years with EBC[3]) = **16·0%.**

Women aged 40–69 years: Chemotherapy

88%* (proportion of all breast cancer that was EBC) × 23% (proportion of women with EBC who received adjuvant chemotherapy[2]) × 33% (EBCTCG: adjuvant chemotherapy reduction in mortality for women aged 40-70 years with EBC[4]) = **6·7%.**

Crude National RMR possible from 1995 adjuvant treatment = 16% + 6.7% = **22·7%**

Measured Crude RMR 1991-2007[5] = 28%

©2020 Burton R and Stevenson C. *JAMA Network Open*.

VICTORIA: all adjuvant therapy 1999 data from Table 2.

Women aged 40–49 years: Tamoxifen:

88%* (proportion of all breast cancer that was EBC) × 73% (proportion of women with EBC who were ER+ and aged less than 50 years-Dr V White-TCCV) × 78% (proportion of women with EBC less than 50 years of age who were ER+ and received Tamoxifen[6]) × 31% (EBCTCG: adjuvant tamoxifen reduction in mortality for ER+ women aged 40-70 years with EBC[3]) = **15·5%.**

Women aged 50–69 years: tamoxifen

88%* (proportion of all breast cancer that was EBC) × 78% (proportion of women with EBC who were ER+ and aged more than 50 years-Dr V white TCCV) × 92% (proportion of women with EBC more than 50 years of age who were ER+ and received Tamoxifen[6]) × 31% (EBCTCG: adjuvant tamoxifen reduction in mortality for ER+ women aged 40-70 years with EBC[3]) = **19·6%.**

Women aged 40–49 years: Chemotherapy

88%* (proportion of all breast cancer that was EBC) × 72% (proportion of women with EBC aged less than 50 years who received adjuvant chemotherapy[6]) × 33% (EBCTCG: adjuvant chemotherapy reduction in mortality for women aged 40-70 years with EBC[4]) = **20·9%.**

©2020 Burton R and Stevenson C. *JAMA Network Open*.

Women aged 50–69 years: Chemotherapy

88%* (proportion of all breast cancer that was EBC) × 29% (proportion of women with EBC aged more than 50 years who received adjuvant chemotherapy[6]) × 33% (EBCTCG: adjuvant chemotherapy reduction in mortality for women aged 40-70 years with EBC[4]) = **8·4%**

Crude RMR possible from 1999 adjuvant treatment

Premenopausal Women (< 50 years) = 20% of 36·4 = 7·3%

Postmenopausal Women (> 50 years) = 80% of 28·0 = 22·4%

Crude RMR possible = 7·3% + 22·4% = **29.7%**

Measured Crude RMR 1994-2013 (Figure 1) = 34·3-23·9/34·3 = **30.3%**

WA: data from Mc Evoy and colleagues[7] and WA breast cancer deaths trend[8]

Women aged 40–69 years: tamoxifen

85%* (proportion of all breast cancer that was EBC) × 68% (proportion of women with EBC who were ER+ and received tamoxifen[7]) × 31% (EBCTCG: adjuvant tamoxifen reduction in mortality for ER+ women aged 40-70 years with EBC[3]) = **15·0%.**

Women aged 40–69 years: Chemotherapy

85%* (proportion of all breast cancer that was EBC) × 44% (proportion of women with EBC who received adjuvant chemotherapy[7]) × 33% (EBCTCG: adjuvant

©2020 Burton R and Stevenson C. *JAMA Network Open*.

chemotherapy reduction in mortality for women aged 40-70 years with EBC[4]) =

12·3%.

Crude WA RMR possible from 1995 adjuvant treatment = 15% + 12.3% = **27·3%**

Population crude breast cancer calculated deaths and incidence trends from the WA Cancer Registry[8] and ABS population reports[9]

1999: deaths 239, female population 0·92m, incidence = 25·9 per 100,000

2014: deaths 249, female population, 1·27m, incidence = 19·6 per 100,000

Measured Crude breast cancer RMR 1999-2014 = 24·3%

©2020 Burton R and Stevenson C. *JAMA Network Open*.

eTable. Data Provided by Vicky Thursfield From VCR

Victorian Breast Cancer Rates 1982-2013				
Year	Vic ASM	VICMortC	VICIncCru	VIC ASI
1982	34.3	31.57	75.82346	86.7
1983	32.5	29.83218	71.73213	81.3
1984	31.4	29.1258	78.51377	88.3
1985	34.4	28.29808	78.89001	87.1
1986	31.8	29.11532	78.94547	87.4
1987	33.2	30.60852	86.07171	94.5
1988	32.5	30.24227	83.78367	91.2
1989	33.5	31.3899	87.87334	94.9
1990	33.4	31.46027	87.98902	94.9
1991	34.9	33.03026	91.95516	98.5
1992	31.3	29.84475	93.8996	99.4
1993	33.3	32.70477	99.48995	103.8
1994	34.5	34.29012	117.5156	120.6
1995	31	31.13485	116.0081	118.1
1996	30.5	30.89614	113.6926	114.5
1997	30.4	31.49536	112.6747	112.1
1998	27.6	28.99101	116.6492	114.3
1999	27.6	29.23969	118.5267	115
2000	27.6	29.82846	121.6599	116.7
2001	25.1	27.38213	123.0128	118.1
2002	26.5	29.4662	121.4662	115.6
2003	27	30.08287	116.809	109.9
2004	24.5	27.65319	122.155	114.3
2005	24.5	27.68691	120.2143	111.1
2006	21.4	24.81846	124.0923	114.2
2007	23.6	25.20722	122.9236	112.5
2008	24.1	28.238	129.3519	117.6
2009	22.4	26.95132	120.9487	109.7
2010	23.1	28.27069	127.2	115
2011	21.6	25.77893	134.1863	120.7
2012	22	26.6341	130.7109	117.3
2013	19.9	23.8799	141.0018	125.3

©2020 Burton R and Stevenson C. *JAMA Network Open*.

eReferences.

1. Hill DJ, Giles GG, Russell IS, Collins JP, Mapperson KJ. Management of primary, operable breast cancer in Victoria. *Med J Aust*. 1990;152(2):67-72.

2. Hill DJ, Jamrozik K, White VM, et al. Surgical management of breast cancer in Australia in 1995, Surgical Management of Breast Cancer in Australia in 1995, NHMRC National Breast Cancer Centre, Kings Cross NSW. Deposited August 1, 1999. http://ecite.utas.edu.au/18185

3. Early Breast Cancer Trialists' Collaborative Group (EBCTCG). Effects of chemotherapy and hormonal therapy for early breast cancer on recurrence and 15-year survival: an overview of the randomised trials. *Lancet*. 2005;365(9472):1687-1717.

4. Peto R, Davies C, Godwin J, Gray R, Pan HC, Clarke M, et al. Comparisons between different polychemotherapy regimens for early breast cancer: meta-analyses of long-term outcome among 100,000 women in 123 randomised trials. *Lancet.* 2012;379:432-44.

5. Burton RC, Bell RJ, Thiagarajah G, Stevenson C. Adjuvant therapy, not mammographic screening, accounts for most of the observed breast cancer specific mortality reductions in Australian women since the national screening program began in 1991. *Breast Cancer Res Treat.* 2012; 131:949-55.

6. White V, Pruden M, Giles G et al. The management of early breast carcinoma before and after the introduction of clinical practice guidelines. Cancer 2004; 101:476-85.

©2020 Burton R and Stevenson C. *JAMA Network Open*.

7. McEvoy SP, Ingram DM, Byrne MJ et al. Breast cancer in Western Australia: clinical practice and clinical guidelines. *Med J Aust.* 2004;181: 305–309.

8. Cancer Council Western Australia. Western Australia cancer statistics, 2014: breast cancer. Posted May 18, 2016. Accessed August 26, 2019. . https://web.archive.org/web/20170301071500/https://www.cancerwa.asn.au/resources/2016-05-18-Breast- cancer-2014-FINAL.pdf

9. Australian Bureau of Statistics 105.0.65.001 - Australian Historical Population statistics, 2014.

©2020 Burton R and Stevenson C. *JAMA Network Open*.
